# Perineal Urethrostomy: Surgical and Functional Evaluation of Two Techniques

**DOI:** 10.1155/2015/365715

**Published:** 2015-02-19

**Authors:** Nicolaas Lumen, Matthias Beysens, Charles Van Praet, Karel Decaestecker, Anne-Francoise Spinoit, Piet Hoebeke, Willem Oosterlinck

**Affiliations:** Department of Urology, Ghent University Hospital, De Pintelaan 185, 9000 Ghent, Belgium

## Abstract

*Introduction*. PU is an option to manage complex and/or recurrent urethral strictures and is necessary after urethrectomy and/or penectomy. PU is generally assumed to be the last option before abandoning the urethral outlet. *Methods*. Between 2001 and 2013, 51 patients underwent PU. Mean age (± standard deviation) was 60 ± 15 years. Only 13 patients (25.5%) did not undergo previous urethral interventions. PU was performed according to the Johanson (*n* = 35) or Blandy (*n* = 16) technique and these 2 groups were compared for surgical failure, maximum urinary flow (Q_max_), urinary symptoms, and quality of life (according to the International Prostate Symptom Score). *Results*. Both groups were similar for patient's and stricture characteristics. Only follow-up duration was significantly longer after Johanson PU (47.9 months versus 11.1 months; *P* = 0.003). For the entire cohort, 11 patients (21.6%) were considered a failure (9 or 25.7% for Johanson group and 2 or 12.5% for Blandy group; *P* = 0.248). There was a significant improvement of Q_max_ in both groups. Quality of life after PU was comparable in both groups. *Conclusions*. PU is associated with a 21.6% recurrence rate and the patient should be informed about this risk.

## 1. Introduction

Urethroplasty is the best option to restore urethral patency in case of urethral stricture disease [1, 2]. Nevertheless, urethroplasty is associated with a failure rate of 10–50%, depending on stricture etiology, stricture length, previous interventions, and the type of technique used [3–6]. Stricture recurrence after (several attempts of) urethroplasty might trigger the decision to stop further attempts in restoring patency of the entire urethra. The surgeon might take this decision because he has no further reconstructive options left or the patient might take this decision because he does not want further reconstruction with the risk of recurrent stricture [7]. At that point, perineal urethrostomy (PU) is a valuable option. A successful PU allows the patient to resume normal voiding and is generally assumed to be the last option before abandoning the urethral outlet. This procedure is reported to be a satisfactory solution, especially in the elderly [7]. PU is also needed after urethrectomy and/or penectomy [8, 9]. Different types of PU have been described [7, 10–12]. These techniques are mainly derived from the first stage of the two-stage urethroplasty described by Johanson [13] and Blandy et al. [14], both renowned pioneers in the field of urethral surgery.

The aim of this paper is to evaluate the surgical and functional outcome after Johanson or Blandy PU. To our knowledge, this is the largest series published on Johanson PU and the first to compare Johanson with Blandy PU.

## 2. Material and Methods

### 2.1. Patient Population

Fifty-one patients underwent PU at the Ghent University Hospital between January, 2001, and June, 2013 (Table 1). Data were retrospectively analysed. Mean (± standard deviation) and median (interquartile range) follow-up of the entire cohort was, respectively, 36 (± 41.6) and 16 (8–48) months. The Johanson and Blandy technique was used in 35 and 16 patients, respectively. Median follow-up was significantly longer in the Johanson group compared to the Blandy group (36 versus 9 months; *P* < 0.001). Early postoperative complications were scored according to the Clavien-Dindo classification [15]. Patients were further followed up on a regular basis with history taking, clinical examination, and uroflowmetry. In case of suspicion of stenosis, urethrography and ureteroscopy were performed. Need for any additional urethral instrumentation (including dilation) was defined as failure. In November 2013, all surviving patients (*n* = 44) were sent the IPSS (International Prostate Symptom Score). The IPSS is used to score obstructive and irritative voiding symptoms with a score varying from 0 (no symptoms) to 35 (very severe symptoms). It also contains a question on quality of life (QoL) varying from 0 (very satisfied) to 6 (very dissatisfied). We redistributed QoL score into 3 groups: satisfactory QoL (score 0 → 2), acceptable QoL (score = 3), and dissatisfied with QoL (score 4 → 6). The study was approved by the local ethics committee (EC UZG 2007/434 and EC UZG 2008/234).

### 2.2. Surgical Technique

**Figure 1:**
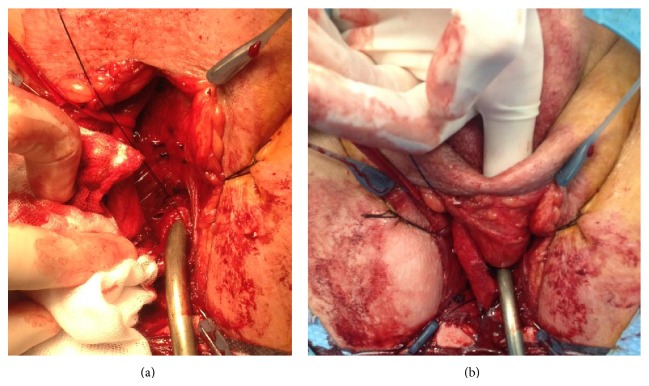
Technique of Johanson PU. After opening of the proximal urethra (a). Mobilisation of the scrotal skin to the proximal urethra (b).

**Figure 2:**
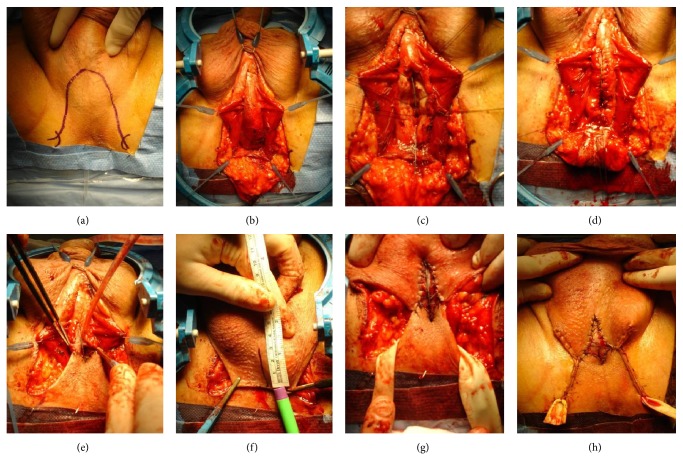
Technique of Blandy PU. Inverted-U perineal incision (a). Exposure of the bulbar urethra (b). Opening of the bulbar urethra (c). Hemostatic sutures on the corpus spongiosum and suturing of the tip of the inverted-U flap towards the proximal urethral opening (d). Suturing of the inverted-U flap to the urethral edges (e). Creation of scrotal flaps (f) and advancement to the urethral mucosa (g). Closure of the wound and final result (h).

Patients were placed in the lithotomy position. For Blandy PU, an inverted-U perineal incision was made. For the Johanson procedure, an inverted-U or midline perineal incision was performed. The bulbar urethra was exposed and opened ventrally. The urethrotomy was extended proximally until healthy urethra was encountered. For patients with stricture, at the membranous urethra, the urethrotomy was extended into the membranous urethra up to the apex of the prostate. In this series, a complete transection of the urethra with mobilization of the proximal urethral stump towards the perineum was never performed. For Johanson PU, scrotal skin was invaginated towards the opened urethra (Figure 1) and an incision was made at the scrotal skin according to the length of the opened urethra. The scrotal skin edges were sutured to the urethral mucosa with interrupted sutures Vicryl 3.0. For an extensive description and illustrations of this technique, we refer to a previous publication [12]. For Blandy PU, the apex of the inverted-U perineal flap was sutured to the most proximal part of the opened urethra. The edges of the perineal flap were further sutured distally to the urethral mucosal edges with Vicryl 3.0. From the moment tension occurred between the sutures, a midline incision was made at the posterior scrotal skin, and two scrotal skin flaps were mobilized to finalize the PU (Figure 2).

### 2.3. Statistical Analysis

Groups were analyzed with independent samples *t*-test (equal variances) or Mann-Whitney test (unequal variances) for continuous variables and with Chi-square or Fischer's exact test for categorical variables. One-year failure free survival (FFS) was evaluated using Kaplan-Meier survival analysis and log-rank statistics. Logistic regression analysis was performed to identify factors predicting failure and QoL. For failure, analyzed factors were patient's age, stricture etiology, stricture length, stricture location, previous interventions, type of operation, and presence of a suprapubic catheter. For QoL, analyzed factors were patient's age, stricture etiology, and failure of PU. A *P* value <0.05 is considered as statistically significant.

## 3. Results and Discussion

Only 13 (25.5%) patients did not undergo previous urethral interventions. Seven (13.7%) patients underwent at least one urethrotomy and/or dilation. Thirty-one patients (60.8%) underwent at least one previous urethroplasty (with or without urethrotomy/dilation). Ten (19.6%) patients underwent PU after urethrectomy: 3 patients because of urethral malignancy and 7 patients concomitant with penectomy for penile malignancy (*n* = 5) or penile gangrene (*n* = 2).

For the entire cohort, 11 (21.6%) patients suffered a failure. In the Johanson group, 9 (25.7%) patients experienced failure versus 2 (12.5%) patients in the Blandy group (*P* = 0.248) (Table 2). One-year FFS was 86.5% for the entire cohort and 87.3 and 84.8% for the Johanson and Blandy groups, respectively (log-rank *P* = 0.904). The mean time to recurrence was 29.7 months for the Johanson group and 5 months for the Blandy group (*P* = 0.415). In the Johanson group, 3 and 2 failures were treated with VY-plasty and intermittent urethrotomies/dilations, respectively. The remaining 4 failures were treated with a Blandy PU, mesh graft augmented PU, buccal mucosa graft augmented PU, and a 7-flap perineal urethrostomy. The failures in the Blandy group were treated with a mesh graft augmented PU. Eight (15.7%) patients suffered a postoperative complication, with 4 complications reported in each group (Johanson 11.5% versus Blandy 25%; *P* = 0.237). One patient developed urosepsis and was treated with intravenous antibiotics (grade 2). Six patients suffered wound dehiscence treated conservatively with secondary healing in 4 patients (grade 1) and by surgical closure in 2 patients (grade 3b). One patient had a severe postoperative bleeding and needed surgical exploration (grade 3b). The two failures in the Blandy group suffered a postoperative wound dehiscence with secondary healing and this was identified as the cause of failure. For the other failures, no apparent cause could be identified. Compared to the preoperative situation, there was a significant improvement of maximum urinary flow (*Q*_max⁡_) (3.1 versus 13.0 mL/s; *P* < 0.001). This improvement remained significant for both groups: from 2.6 to 10.9 mL/s in the Johanson group and from 3.9 to 16 mL/s in the Blandy group.

Of 44 surviving patients, 32 (62.7%) patients sent back the questionnaires (19 and 13 patients after Johanson and Blandy PU, resp.). One patient treated with Johanson PU only answered the QoL questionnaire. Mean postoperative IPSS was 8.7 for the entire cohort. IPSS after Johanson and Blandy PU was, respectively, 10.2 and 6.6 (*P* = 0.078). For the entire cohort, 14 (43.7%), 12 (37.5%), and 6 (18.8%) patients reported, respectively, a satisfactory, acceptable, and dissatisfactory QoL after PU. Four dissatisfied patients had Johanson PU (21.1%) and two had Blandy PU (15.4%). Differences in QoL were not statistically significant between both groups (*P* = 0.635).

Logistic regression analysis could not identify any significant factors that predicted failure of PU (Table 3). Failure of PU tends to predict dissatisfaction with QoL (odds ratio 5.5; 95% CI 0.8–37.6; *P* = 0.08).

PU is an option to manage complex and/or recurrent urethral strictures [7, 12, 16] and is needed after urethrectomy/penectomy [8, 9]. Nevertheless, success with PU is far from guaranteed as shown by the 21.6% failure rate in this series. This failure rate corresponds well to the 0–30% failure rate reported by other series [7, 10, 11, 16, 17]. Comparison between series is difficult because of differences in follow-up and study population. Peterson et al. reported 100% success [16]. In this series, only 18% of cases underwent prior urethroplasty and stricture etiology was lichen sclerosus which mainly affects the penile urethra. Therefore, PU could be performed at a healthy, unaffected bulbar urethra. These factors probably explain the favorable results in their series. This is in contrast to our series and the series of Barbagli et al. [7], Kulkarni et al. [17], and Myers et al. [11], where, respectively, 60.8%, 52.6%, 96.3%, and 48% of patients underwent one or more urethroplasties. Previous urethroplasty is a well-known risk factor for failure in urethral reconstruction [3, 5, 6]. Indeed, after previous urethroplasty, the urethra is already scarred and of poorer quality and if this segment needs to be used for PU, it can lead to further scarring and narrowing of PU. This is certainly the case in our series where only 23.5% of patients had no involvement of the bulbar urethra, which is incorporated in the PU. This might explain the higher failure rate in our series and the series of Barbagli et al. [7], Kulkarni et al. [17], and Myers et al. [11] In this context, it is important to inform the patient that recurrence after PU is possible, despite the fact that it is offered as the final solution for his complex/recurrent stricture.

Until 2009, Johanson PU was our preferred technique. From 2010, we started with Blandy PU as preferred modality. This has several reasons. (1) In 2009, two major series were published on Blandy PU and currently Blandy PU is the standard technique for PU. So, we started to follow the mainstream in PU, which explains the significant shorter follow-up time with Blandy PU. (2) The Johanson technique has several inconveniences for the patient: the invagination of the scrotal skin is unaesthetic and the patient urinates on the scrotum (Figure 1). (3) We find the Blandy technique easier to perform compared to the Johanson technique. This might be contradictory as the operation time with Blandy technique is longer compared to the Johanson technique. This longer operation time may reflect the learning curve we had to go through with the Blandy technique. Furthermore, during Blandy PU technique, we sutured the edges of the opened corpus spongiosum for hemostasis (Figure 2(d)), which was not performed during Johanson PU. This was the reason for one case of the severe postoperative bleeding in the Johanson group. Because of the significant difference in follow-up for the Johanson and Blandy groups, it is not possible to draw conclusions about possible differences or equalities between both techniques based on these data. However, Blandy PU is now generally considered as the standard technique for PU, and our data suggest that Johanson PU remains an option, especially when Blandy PU is not possible. This is, for instance, the case if the bulbar urethra is approached by a midline perineal incision for urethroplasty and when the peroperative findings make a one-stage reconstruction impossible. In this case, a two-stage procedure or formal PU must be performed. A Blandy PU is no longer possible, since no inverted-U flap is available, but the Johanson technique is still an option. An alternative for this case is a 7-flap perineal urethrostomy [10]. Another case is when the inverted-U flap of the Blandy technique cannot be brought towards the proximal urethra without any tension (e.g., obese patients or stricture extending in the proximal bulbar urethra or membranous urethra). As the scrotal skin is very elastic, it can usually be mobilized even up to the level of the membranous urethra, and, for this case, the Johanson technique can be performed. In our series, both failures in the Blandy group were due to tension at the anastomosis between the urethra and the inverted-U flap with wound dehiscence and subsequent stenosis of the neomeatus. These failures might have been prevented if another technique such as the Johanson technique would have been used. Another option would be to transect the urethra and mobilize the proximal urethral stump towards the perineal incision. This was never performed in this series. Particularly for membranous or deep bulbar strictures, this maneuver will not lead to a substantial gain of length in order to reduce tension with perineal skin flaps. Furthermore, transection of the urethra will eliminate the retrograde blood supply of the proximal urethra. As the majority of patients in our series already underwent previous urethroplasty, we opted to preserve the urethral blood supply as much as possible to avoid ischemic damage to the urethra at which the PU is performed. Myers et al. [11] also postulated that preservation of the urethral blood supply is an important key to success in PU.

In this series, 18.8% of patients were dissatisfied after PU with no significant differences between the two groups. This is higher compared to the 1.2% and 0% dissatisfaction rate in the series of Barbagli et al. [7] and Peterson et al. [16], respectively. Failure of PU tends to predict dissatisfaction. This might explain our higher dissatisfaction rate compared to the series of Peterson et al. [16] where no patients suffered a recurrence. This, however, cannot explain the higher dissatisfaction rate compared to the series of Barbagli et al. [7] with a similar failure rate. For 3 out of 6 dissatisfied patients, there was a specific reason in this series. In one patient, PU was the end point of an iatrogenic stricture after transurethral resection of the prostate. He considered this a serious complication of what he expected to be a simple procedure for benign prostatic hyperplasia [18]. This patient was more dissatisfied with the whole urologic history rather than with PU itself. Two other dissatisfied patients underwent PU after penectomy. In these patients, PU was not a choice but a necessity that was suddenly needed. This is in contrast to patients who have a long history of urethral stricture disease in which PU is often a relief. Most of these patients in our series indeed underwent several urethral manipulations (urethroplasty, urethrotomy, and dilations) or had a suprapubic catheter. For these patients it is a relief to be able to void again, even if this is in a seated position. It is much more likely that these patients will be more satisfied with PU compared to patients after urethrectomy/penectomy who are “condemned” to PU.

This study has several important limitations. It concerns a single-center and retrospective study. The follow-up duration differs significantly between both groups and is on average less than 1 year in the Blandy group. A comparison between both groups is therefore subject to important bias and hard conclusions cannot be made based on these data. With longer follow-up, it is probable that more failures will be detected in the Blandy group, further affecting surgical and functional outcome. Evaluation of the functional outcome with questionnaires is hampered by 37% of patients who did not return the questionnaire. It is impossible to estimate whether or not more dissatisfied patients have not responded to the invitation to fill in the questionnaire. IPSS is used to score obstructive and irritative symptoms after PU. Although IPSS is frequently used to describe voiding symptoms before and after urethral reconstruction, it is not validated for this pathology and Nuss et al. [19] reported that 21% of patients with urethral stricture disease reported symptoms that were not scored by IPSS. Validated questionnaires for urethral reconstruction are recently developed [20] and should be used in the future for further evaluation. Another limitation is that there are no functional scores available before PU nor is there a control group that remained on suprapubic diversion or repetitive urethral manipulations.

## 4. Conclusion

About 1 out of 5 patients suffered a recurrence after PU and the patient should be informed about this risk. Although the majority of patients had a satisfactory/acceptable QoL after PU, the dissatisfaction rate in this series is higher than previously reported.

## Figures and Tables

**Table 1:** Patient and stricture characteristics. Values are presented as mean ± standard deviation or number (%). For follow-up, the median value with interquartile range is provided because of unequal variances.

	Total (*n* = 51)	Johanson (*n* = 35)	Blandy (*n* = 16)	*P* value
Follow-up (months)				
Mean (± standard deviation)	36.3 ± 41.6	47.9 ± 45.5	11.1 ± 10.4	**0.002** ^*^
Median (interquartile range)	16 (8–48)	36 (11–75)	9 (6–13)	<**0.001** ^*^
Age (years)	60.1 ± 15.1	60.5 ± 14.7	59.2 ± 16.4	0.769°
Stricture length (cm)	8.6 ± 5.0	9.3 ± 5.0	7.1 ± 4.8	0.141°
Preop *Q*_max⁡_ (mL/s)	3.1 ± 4.8	2.6 ± 3.1	3.9 ± 6.6	0.483°
Etiology				
Idiopathic	8 (15.7%)	6 (17.1%)	2 (12.5%)	
Iatrogenic	21 (41.2%)	17 (48.6%)	4 (25.0%)	
Traumatic	5 (9.8%)	2 (5.7%)	3 (18.8%)	0.271^§^
Inflammatory	7 (13.7%)	5 (14.3%)	2 (12.5%)	
Urethrectomy	10 (19.6%)	5 (14.3%)	5 (31.2%)	
Previous interventions				
None	13 (25.5%)	8 (22.96%)	5 (31.2%)	
DVIU/dilation	7 (13.7%)	7 (20.0%)	0 (0.0%)	0.074^§^
Urethroplasty	31 (60.8%)	20 (57.1%)	11 (68.8%)	
Location				
Bulbar	12 (23.5%)	10 (28.6%)	2 (12.5%)	
Penile	12 (23.5%)	7 (20.0%)	5 (31.2%)	
Membranous	5 (9.8%)	1 (2.9%)	4 (25.0%)	0.053^§^
Panurethral	22 (43.1%)	17 (48.6%)	5 (31.2%)	
Suprapubic catheter				
No	35 (68.6%)	24 (68.6%)	11 (68.8%)	
Yes	16 (31.4%)	11 (31.4%)	5 (31.2%)	0.99^#^

(^*^Mann-Whitney test; °independent samples *t*-test; ^§^Fisher's exact test; ^#^Chi-square test.)

**Table 2:** Surgical and functional outcomes. Values are presented as mean ± standard deviation or number (%).

	Total	Johanson	Blandy	*P* value
Operation time (min)	102.1 ± 37.3	97.2 ± 33.7	112.6 ± 43.4	0.172°
Failure				
No	40 (78.4%)	26 (74.3%)	14 (87.5%)	0.248^§^
Yes	11 (21.6%)	9 (25.7%)	2 (12.5%)	
Time to recurrence (months)	31.6 ± 39	29.7 ± 39.2	5.0 ± 2.8	0.415°
Complications				
No	43 (84.3%)	31 (88.6%)	12 (75.0%)	
Grade 1	4 (7.8%)	1 (2.9%)	3 (18.8%)	0.237^§^
Grade 2	1 (2.0%)	1 (2.9%)	0 (0%)	
Grade 3	3 (5.9%)	2 (5.7%)	1 (6.2%)	

	*n* = 26	*n* = 15	*n* = 11	

Postop *Q*_max⁡_ (mL/s)	13.0 ± 7.5	10.9 ± 6.3	16.0 ± 8.2	0.087°

	*n* = 31	*n* = 18	*n* = 13	

IPSS (0 → 35)	8.7 ± 5.6	10.2 ± 5.1	6.6 ± 5.8	0.078°
Postvoid dribbling				
Absent	23 (74.2%)	14 (77.8%)	9 (69.2%)	
Present	8 (25.8%)	4 (22.2%)	4 (30.8%)	0.448^§^

	*n* = 32	*n* = 19	*n* = 13	

QoL				
Satisfied (0 → 2)	14 (43.7%)	7 (36.8%)	7 (53.8%)	
Acceptable (3)	12 (37.5%)	8 (42.1%)	4 (30.8%)	0.635^§^
Dissatisfied (4 → 6)	6 (18.8%)	4 (21.1%)	2 (15.4%)	

(°Independent samples *t*-test; ^§^Fisher's exact test.)

**Table 3:** Univariate logistic regression analysis.

Predictive factor for failure	OR (95%-CI)	*P* value
Type of operation	0.179 (0.019–1.658)	0.13
Follow-up	1.007 (0.991–1.023)	0.405
Age	0.984 (0.937–1.034)	0.525
Stricture length	1.099 (0.945–1.279)	0.221
Etiology	0.957 (0.530–1.729)	0.885
Previous interventions	0.653 (0.247–1.729)	0.391
Location	0.839 (0.549–2.091)	0.839
Suprapubic catheter	0.243 (0.026–2.272)	0.215
